# Phosphorylation induces distinct alpha-synuclein strain formation

**DOI:** 10.1038/srep37130

**Published:** 2016-11-17

**Authors:** Meng-Rong Ma, Zhi-Wen Hu, Yu-Fen Zhao, Yong-Xiang Chen, Yan-Mei Li

**Affiliations:** 1 Department of Chemistry, Key Laboratory of Bioorganic Phosphorus Chemistry and Chemical Biology (Ministry of Education), Tsinghua University, Beijing 100084, P. R. China; 2 Beijing Institute for Brain Disorders, Beijing 100069, P. R. China

## Abstract

Synucleinopathies are a group of neurodegenerative diseases associated with alpha-synuclein (α-Syn) aggregation. Recently, increasing evidence has demonstrated the existence of different structural characteristics or ‘strains’ of α-Syn, supporting the concept that synucleinopathies share several common features with prion diseases and possibly explaining how a single protein results in different clinical phenotypes within synucleinopathies. In earlier studies, the different strains were generated through the regulation of solution conditions, temperature, or repetitive seeded fibrillization *in vitro*. Here, we synthesize homogeneous α-Syn phosphorylated at serine 129 (pS129 α-Syn), which is highly associated with the pathological changes, and demonstrate that phosphorylation at Ser129 induces α-Syn to form a distinct strain with different structures, propagation properties, and higher cytotoxicity compared with the wild-type α-Syn. The results are the first demonstration that post-translational modification of α-Syn can induce different strain formation, offering a new mechanism for strain formation.

The presence of alpha-synuclein (α-Syn) inclusions in neurons and glia of the central nervous system is associated with a group of neurodegenerative diseases termed synucleinopathies, including Parkinson’s disease (PD), dementia with Lewy bodies (DLB), and multiple-system atrophy (MSA)^1^. However, the synucleinopathies are strikingly distinct clinical syndromes; subcortical and cortical Lewy bodies (LBs) are the hallmark of PD and DLB while glial α-Syn deposits are the main characteristic of MSA^2^.

Increasing evidence supports the hypothesis that α-Syn aggregates spread within the nervous system in a prion-like fashion. α-Syn-positive LBs propagate from brains of patients with PD to the grafted cells^3^,^4^, and intracerebral injection of sarkosyl-insoluble α-Syn from brains of patients with DLB induces accumulated α-Syn pathology in wild-type mice^5^. Moreover, small amounts of exogenously preformed α-Syn fibrils can act as seeds to recruit endogenous intracellular α-Syn to aggregate and transmit cell to cell in cultured cells and living animals^6^,^7^,^8^ in a manner similar to prion protein propagation. Additionally, the presence of α-Syn strains, which is a typical characteristic of prion diseases, has been described^9^,^10^,^11^. Via repetitive seeded fibrillization *in vitro*, Lee *et al.* discovered two distinct strains of α-Syn with different abilities to cross-seed tau aggregation in cultured neurons and *in vivo*^9^. Melki *et al.* achieved different α-Syn strains through the regulation of solution conditions and demonstrated that α-Syn strains with different structural characteristics and seeding propensities produce distinct histopathological and behavioral phenotypes^10^,^11^. Furthermore, prions from MSA extracts induce the aggregation of α-Syn*A53T-YFP (α-Syn with A53T mutation tagged with yellow fluorescent protein) in cultured cells while PD extracts do not, indicating that MSA is caused by a unique α-Syn strain that differs from the strain causing PD^12^,^13^. Collectively, these reports support the existence of strains, which could explain the variability of different clinical phenotypes within synucleinopathies.

What may affect different strain formation *in vivo*? The main component of the pathological lesions is extensively phosphorylated α-Syn at serine 129 (pS129 α-Syn)^14^, and pS129 α-Syn may play a critical role in synucleinopathy pathogenesis. Phosphorylation at Ser129 can regulate α-Syn fibril formation^14^,^15^,^16^,^17^ and enhance α-Syn toxicity both *in vitro* and *in vivo*^18^,^19^,^20^,^21^. As the main form of α-Syn in the pathological process, pS129 α-Syn may be associated with multiple strain formation *in vivo*. To confirm the effect of phosphorylation at Ser129 on α-Syn strain formation, we took advantage of expressed protein ligation to semisynthesize the homogeneous pS129 α-Syn. Here, we show that phosphorylation at Ser129 can induce α-Syn to form a distinct strain with structural variation, different propagation properties, and higher cytotoxicity compared with the wild-type α-Syn (WT α-Syn), respectively called the PS and WT strains. Our studies provide the first evidence that the post-translational modifications (PTMs) of α-Syn can induce different strain formation, which suggests that PTM status of α-Syn in the pathogenetic process may have an important effect on strain formation.

## Results

### Chemical synthesis and purification of pS129 α-Syn

**Figure 1:**
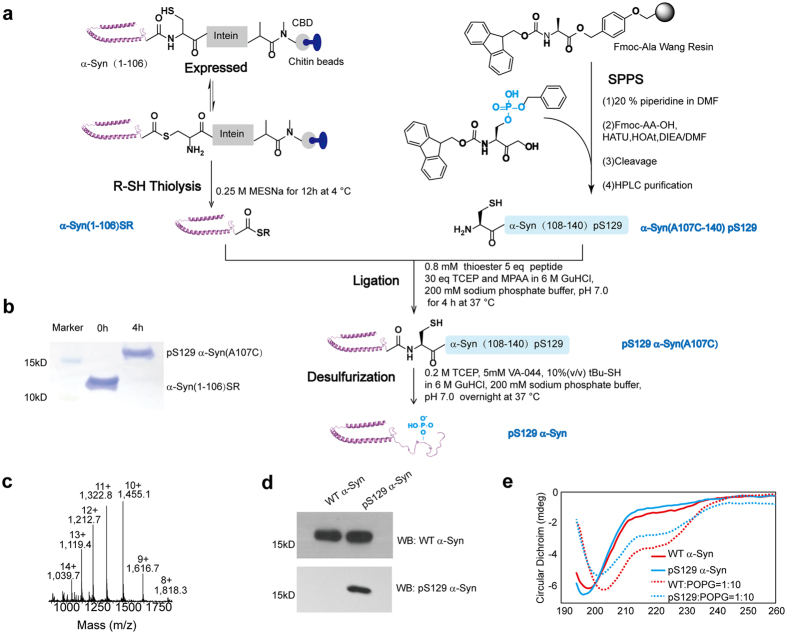
Preparation and folding analysis of pS129 α-Syn. (**a**) Semisynthesis strategy for the preparation of pS129 α-Syn. The scheme shows how recombinant α-Syn(1–106)SR was generated and how pS129 α-Syn was prepared via ligation and desulfurization. (**b**) SDS-PAGE analysis of the generation of pS129 α-Syn(A107C). After 4 h, the α-Syn(1–106)SR was converted to pS129 α-Syn(A107C) completely. (**c**) ESI-MS of pS129 α-Syn was performed to confirm the identity of the protein after desulfurization. The observed mass of 14,541.0 Da is consistent with the calculated mass of 14,538.1 Da. (**d**) Western blot analysis of WT α-Syn and pS129 α-Syn. The primary antibodies are indicated at the right side of each blot. (**e**) Characterization of WT α-Syn and pS129 α-Syn by CD in the absence or presence of lipid vesicles composed of POPG, in which are shown WT α-Syn (solid line, red), pS129 α-Syn (solid line, blue), WT α-Syn:POPG = 1:10 (dashed line, red), and pS129 α-Syn:POPG = 1:10 (dashed line, blue).

Verifying the effect of phosphorylation at Ser129 on α-Syn strain formation requires a chemically well-defined homogeneous pS129 α-Syn. Previously work prepared pS129 α-Syn with the assistance of protein kinase, such as casein kinase 1 and polo-like kinase 2. Iwatsubo *et al.* prepared pS129 α-Syn using casein kinase 1 to phosphorylate recombinant WT α-Syn and found phosphorylation promotes fibril formation^14^. Lashuel *et al.* used the same method to prepare phosphorylated α-Syn with the S87A mutation in case the phosphorylation of Ser87, and the pS129 α-Syn (S87A) inhibited the fibrillization of α-Syn^15^. While Engelborghs *et al.* found that on the strength of polo-like kinase 2 to target Ser129 specifically, pS129 exhibit no influence on fibrillization kinetics of α-Syn^16^. Recently, co-expression of α-Syn with polo-like kinase 2 in *E. coli*, was used to prepare pS129 α-Syn, with resulting earlier fibril formation of pS129 α-Syn compared to WT α-Syn^17^. The inconsistency of the outcome may partially result from the S87A mutation and the efficiency of the enzyme. Expressed protein ligation can help overcome this limitation, involving a native chemical ligation (NCL) reaction between the N-terminal recombinant thioester protein and C-terminal site-specific modified peptide using solid phase peptide synthesis (SPPS) (Fig. 1a). Because the native sequence of α-Syn does not contain any cysteine residues, which are necessary for the NCL reaction, we chose to mutate an alanine to cysteine as the ligation site for the NCL reaction, followed by a return of alanine via desulfurization reaction. Ala124 was not chosen as the ligation site considering that the NCL reaction occurred with glutamic acid (Glu123), which was reported to produce a certain amount of byproducts^22^. We chose Ala107 as the ligation site.

Briefly, α-Syn(1–106) was expressed in fusion with intein and a chitin binding domain (CBD) as the purification tag to facilitate immobilization on the chitin beads. The undesired protein was removed, and the recombinant protein was attached to the beads. The α-Syn(1–106)SR was generated by thiolysis with 0.25 M sodium 2-mercaptoethanesulfonate (MESNa) for 12 h at 4 °C. The α-Syn(1–106)SR was characterized by SDS-PAGE and ESI-MS (Supplementary Fig. S1A). The peptide fragment α-Syn(A107C–140) pS129 was synthesized by stepwise SPPS, and a Fmoc-Ser[PO(OBzl)]-OH building block was used to bring in the phosphorylated serine at 129. The α-Syn(A107C–140) pS129 was purified with RP-HPLC and characterized by analytical RP-HPLC and ESI-MS (Supplementary Fig. S1B). The full-length pS129 α-Syn(A107C) (Fig. 1b) was then prepared via the addition of α-Syn(1–106)SR to the excessive α-Syn(A107C–140) pS129 for 4 h at 37 °C. Ligation between the thioester protein and the peptide fragment was carried out at pH 7.0 in 6.0 M guanidine hydrochloride (GuHCl), 0.2 M sodium phosphate in the presence of 30 mM tris(2-carboxyethyl)phosphine (TCEP), and 50 mM 4-mercaptophenylacetic acid (MPAA) as the NCL catalyst. Afterwards, the ligation mixture was desalted through a 5 mL HiTrap desalting column with 6.0 M GuHCl and 0.2 M sodium phosphate, pH 7.0, as the mobile phase to remove the MPAA. The ligation product pS129 α-Syn(A107C) was characterized by ESI-MS after desalting with water as the mobile phase (Supplementary Fig. S1C). The fraction containing the ligation product was then subjected to a desulfurization reaction using free-radical-based conditions [TCEP, 2,2′-Azobis[2-(2-imidazolin-2-yl)propane]dihydrochloride (VA-044), and 2-methylpropane-2-thiol] overnight at 37 °C and purified with RP-HPLC. PS129 α-Syn was readily generated in multimilligram amounts and isolated in a highly pure form, as confirmed by RP-HPLC, SDS-PAGE, and ESI-MS (Supplementary Fig. S1D, Fig. 1c).

The recombinant WT α-Syn was expressed and purified as Wang *et al.* demonstrated previously^23^. The WT α-Syn was characterized by SDS-PAGE, RP-HPLC, and ESI-MS (Supplementary Fig. S1E). Western blot analysis using anti-pS129 α-Syn and anti-α-Syn antibodies was performed to examine the identity and purity of the semisynthetic pS129 α-Syn and recombinant WT α-Syn (Fig. 1d). The secondary structure of the semisynthetic pS129 α-Syn was investigated by CD spectroscopy in solution (phosphate-buffered saline; PBS) and in the presence of 1-palmitoyl-2-oleoyl-sn-glycero-3-phosphorylglycerol (POPG) vesicles with a lipid-protein mole ratio of 10:1. The CD spectrum of pS129 α-Syn was indistinguishable from that of recombinant WT α-Syn in solution, showing that both proteins exist predominantly in random coil conformations (Fig. 1e). Upon binding to lipid vesicles, both proteins adopted an α-helical structure, but the α-helix of pS129 α-Syn signal was weaker than that of WT α-Syn, indicating that phosphorylation at Ser129 may reduce the membrane binding properties of α-Syn (Fig. 1e).

### Distinct structure of WT and PS fibers

**Figure 2:**
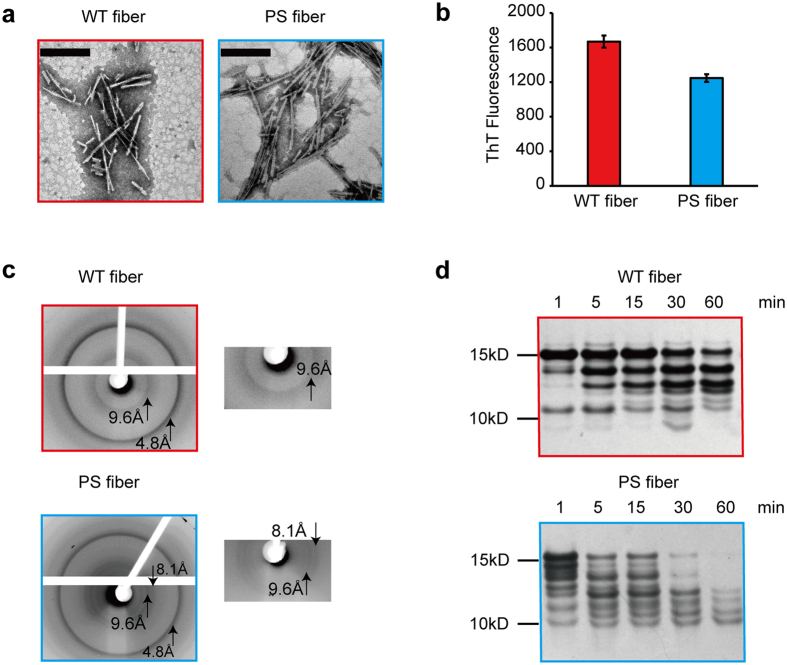
Structural characterization of WT fiber and PS fiber. (**a**) TEM analyses of WT fiber (left) and PS fiber (right) after incubation for 1 week. (Scale bars: 200 nm.) (**b**) ThT fluorescence intensity of WT fiber and PS fiber. After 1 week of incubation, the ThT fluorescence was measured via mixing α-Syn fibers with the ThT solution, and the final concentrations of ThT and α-Syn fibers were 10 μM and 3.5 μM, respectively. (**c**) X-ray diffraction pattern of WT fiber (upper) and PS fiber (lower).Both fibers showed a typical cross-β diffraction pattern containing the characteristic reflections at 4.8 Å and 9.6 Å. Partial enlarged details are presented at the right of each pattern. The PS fiber had an additional reflection at 8.1 Å. (**d**) Proteolytic digestion patterns of WT (upper) and PS (lower) α-Syn fibers (30 μM according to monomer concentration) with 0.1 μg/mL PK at 25 °C, monitored over time (1 h) with silver-stained SDS-PAGE.

Ser129 localizes at the C-terminal domain of α-Syn, instead of the hydrophobic NAC (non-Aβ component of Alzheimer’s disease amyloid) domain (residues 61–95), which is critical for the aggregation of α-Syn. However, C-terminal of α-Syn has long-range interactions with N-terminal and shields the NAC region, which stabilize its conformation and inhibit spontaneous aggregation^24^. It is compatible with the findings that C-terminally truncated α-Syn can accelerate fibrillization of α-Syn^25^. Also, the observation of phosphorylation at Ser129 affects the kinetics of α-Syn fibril formation has been demonstrated^14^,^15^,^16^,^17^, but remains controversial whether phosphorylation promotes or prevents aggregation. As we discussed before, the contradictory results are partially due to the selectivity of the kinase which was used during the preparation of phosphorylated α-Syn. Here, as the well-defined and homogenous pS129 α-Syn has been obtained, we investigated the role of phosphorylation at Ser129 on the kinetics of α-Syn aggregation. With Thioflavin T (ThT) fluorescence assay we monitored the aggregation kinetics of WT α-Syn and pS129 α-Syn (40 μM) at 37 °C under constant agitation conditions. The result showed that pS129 α-Syn fibrillized readily after about 11 h, while WT α-Syn started to fibrillize after more than 24 h (Supplementary Fig. S2), which illustrated that phosphorylation at Ser129 promote α-Syn aggregation. In addition, little is known about the structural characteristics of the fiber formed by pS129 α-Syn. To investigate whether phosphorylation at Ser129 influences the structural diversity of fibrillar α-Syn, WT α-Syn and pS129 α-Syn (70 μM) were incubated in PBS with constant agitation at 37 °C for 1 week. Transmission electron microscopy (TEM) indicated that both WT α-Syn and pS129 α-Syn formed mature fibrils (WT fiber, PS fiber, respectively) and had a similar morphology (Fig. 2a). Both fibers possessed distributions of self-association and twist. However, we found that the WT fiber exhibited higher binding affinities with the amyloid-binding dye ThT compared to the PS fiber, suggesting some structural differences in the two fiber conformations (Fig. 2b).

To probe the architecture of the two fibers, we performed X-ray diffraction and found that both fibers showed a typical cross-β diffraction pattern containing the characteristic 4.8 Å signal corresponding to the distance between β-strands along the fiber axis and the 9.6 Å signal corresponding to the face-to-face distance of the β-sheets (Fig. 2c). There was an additional 8.1 Å peak for the PS fiber, suggesting that it contains two intersheet distances (Fig. 2c). To further examine structural differences between the two fibers, we treated WT and PS fibers with proteinase K (PK) at 25 °C for the indicated time and, as shown by silver-stained SDS-PAGE, the two fibers had distinct PK digestion patterns (Fig. 2d). The remaining full-length pS129 α-Syn was less abundant than WT α-Syn, indicating that the PS fiber was less resistant to the PK. Taken together, these results demonstrated that the WT and pS129 α-Syn assembled into different amyloid conformations with distinct physical properties.

### Higher cytotoxicity induced by PS fiber

**Figure 3:**
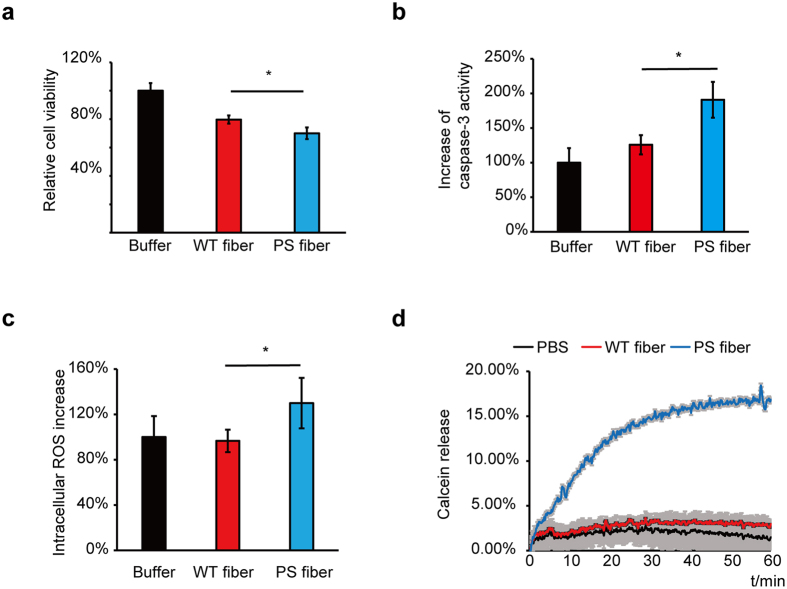
Toxicity of WT fiber and PS fiber. (**a**) Cell viability of SH-SY5Y cells measured by MTT assay. SH-SY5Y cells were treated with buffer (black bar), 1 μM WT fiber (red bar), or 1 μM PS fiber (blue bar) for 24 h. (**b**) Caspase-3 activation in SH-SY5Y cells. SH-SY5Y cells were treated with buffer (black bar), 1 μM WT fiber (red bar), or 1 μM PS fiber (blue bar) for 24 h. Caspase-3 activation was measured with Caspase 3 Activity Assay Kit. (**c**) Intracellular ROS levels in SH-SY5Y cells. SH-SY5Y cells were treated with buffer (black bar), 1 μM WT (red bar), or 1 μM PS (blue bar) α-Syn fibers for 1 h and loaded with 2.5 μM 2′,7′-dichlorofluorescein diacetate (DCFH-DA) for 30 min. The fluorescence was measured with a plate reader. (**d**) Calcein release from POPG lipid vesicles induced by 10 μM WT (red curve) or 10 μM PS (blue curve) α-Syn fibers. Identical volumes of assembly buffer were added to the lipid vesicles to be used as control (black curve). The calcein-loaded lipid vesicles were treated with α-Syn fibers, and fluorescence was monitored for 1 h. In Fig. 3, values for WT and PS fibers are mean ± SD shown as percentages relative to controls. In (**a**,**c**), n = 6 independent measurements; in (b,d), n = 3 independent measurements. Statistical significance was determined by one-way ANOVA, *P < 0.05. (**a**)P = 0.0101, α-value: 0.05; (**b**) P = 0.0320, α-value: 0.050; (**c**) P = 0.0111, α-value: 0.050.

α-Syn under different solution conditions assembles into two different populations with structural variation, resulting in differential cytotoxicity^10^. To explore whether WT and PS fibers induce differential cytotoxicity, the fibers (1 μM based on the monomer) were applied extracellularly to human neuroblastoma SH-SY5Y cells, and cell viability was assessed with the 3-(4,5-dimethylthiazol-2-yl)-2,5-diphenyltetrazolium bromide (MTT) assay. After 24 h of treatment, the PS fiber showed significantly higher cytotoxicity (Fig. 3a). A similar effect was also observed with the addition of the fibers to mouse neuroblastoma N2a cells (Supplementary Fig. S3A).

We also measured the caspase-3 activity of SH-SY5Y with exposure to the two fibers. The PS fiber induced the activation of caspase-3 to a greater extent than did the WT fiber (Fig. 3b), suggesting that the PS fiber was more efficient in triggering cell apoptosis. To further explain the cause of the cytotoxicity, we measured the intracellular oxidative stress and the membrane leakage with treatment with the two fibers. The oxidative stress in the SH-SY5Y cells was assessed with reactive oxygen species (ROS) levels induced by the two fibers. After incubation with each of the two fiber types for 1 h, the PS fiber induced higher ROS accumulation in comparison with the WT fiber (Fig. 3c). We then examined the ability of WT and PS fibers to disrupt membrane integrity using a calcein release assay. Lipid vesicles composed of POPG were capsuled with calcein (70 mM), and calcein release was monitored over time upon exposure to 10 μM WT fibers and PS fibers, respectively. The PS fiber permeabilized lipid vesicles to a much greater extent according to the fluorescence increase with the levels of calcein release (Fig. 3d). All of these observations strongly suggest that the PS fiber triggered higher cytotoxicity and apoptosis, partially involving oxidative stress and membrane destabilization.

### Propagation of WT and PS fibers *in vitro*

**Figure 4:**
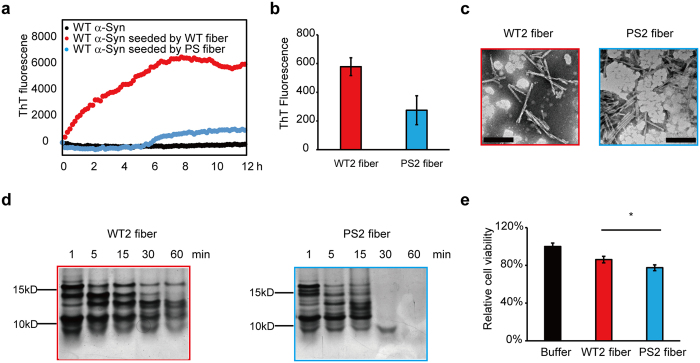
Propagation capacities of WT fiber and PS fiber *in vitro*. (**a**) The kinetics of aggregation of WT monomeric α-Syn (35 μM) in the absence of preformed fiber (black dot) or presence of WT (red dot) or PS (blue dot) α-Syn fibers (3.5 μM based on monomer) monitored by ThT fluorescence. Data were obtained by subtracting the identical concentration of ThT solution. (**b**) ThT fluorescence intensity of WT2 and PS2 α-Syn fibers. WT monomeric α-Syn (70 μM) was incubated with 10% WT or PS fibers (7 μM based on monomer) for 1 week with constant agitation to form WT2 (red bar) and PS2 (blue bar) α-Syn fibers. After 7 days of incubation, ThT fluorescence were performed. (**c**) TEM analyses of WT2 fiber (left) and PS2 fiber (right) after incubation for 1 week. (Scale bars: 200 nm.) (**d**) Proteolytic digestion patterns of WT2 (left) and PS2 (right) α-Syn fibers with PK, monitored over time (1 h) with silver-stained SDS-PAGE. (**e**) Cell viability of SH-SY5Y cells treated with buffer (black bar), 1 μM WT2 α-Syn fiber (red bar), or 1 μM PS2 α-Syn fiber (blue bar) for 24 h, measured by MTT assay. Values for WT2 and PS2 fibers are mean ± SD (n = 6 independent measurements) shown as percentages relative to control. Statistical significance was determined by one-way ANOVA, *P < 0.05. P = 0.0104, α-value: 0.050.

To confirm whether WT and PS fibers can recruit the monomeric α-Syn, we monitored the kinetics of aggregation of WT α-Syn (35 μM) in the presence of 3.5 μM (based on monomer) of the two preformed fibers at 37 °C under constant agitation conditions. In the absence of the preformed fibers, no increase in ThT fluorescence signal was observed in 12 h, indicating no detectable aggregation of α-Syn (Fig. 4a). In the presence of the WT fiber as the seed to induce aggregation, the ThT fluorescence signal increased rapidly with no lag phase, suggesting that the preformed WT fiber was strongly able to recruit the monomeric α-Syn (Fig. 4a). In contrast, upon seeding with the PS fiber, the aggregation of monomeric α-Syn still had a lag time of about 6 h, indicating that the PS fiber had a weaker capacity to seed the monomeric WT α-Syn compared to the WT fiber (Fig. 4a). To identify which fiber can completely dominate the aggregation reaction, 3.5 μM (based on monomer) of both fibers were co-incubated with 35 μM soluble α-Syn at 37 °C under quiescent condition and the aggregation kinetics was monitored with ThT fluorescence assay. Besides, soluble monomeric α-Syn without preformed fiber or incubated with one of the two fibers was used as control. We observed that α-Syn aggregation rate co-incubated with both WT and PS fibers is similar with in the presence of WT fiber alone, while PS fiber did not seed α-Syn aggregation in 12 h under the quiescent condition (Supplementary Fig. S4), indicating that WT fiber can dominate the aggregation process of PS fiber.

To evaluate whether the two fibers could faithfully propagate the structural characteristics to the monomeric α-Syn, WT α-Syn monomers (70 μM) were incubated in PBS seeded by 10% WT fiber and PS fiber (7 μM based on monomer), respectively, with constant agitation at 37 °C for 1 week. TEM showed that the WT and PS fibers both induced α-Syn to form mature fibrils (WT2 fiber, PS2 fiber, respectively) and that their morphology was still similar (Fig. 4c). Both fibers were self-associated and twisted. Upon measurement of the ThT fluorescence of the WT2 fiber and PS2 fiber, we found that the ThT fluorescence signal of the WT2 fiber was higher, suggesting that the WT2 fiber exhibited higher binding affinities with ThT than did the PS2 fiber (Fig. 4b). This result showed the same trend with the preformed WT fiber and PS fiber, suggesting a similar structural property with the preformed fibers (Fig. 2b). Limited proteolysis further showed that the PS2 fiber was less resistant to PK than was the WT2 fiber (Fig. 4d), mirroring the characteristics of the WT and PS fibers (Fig. 2d). The clear indication is that WT and PS fibers can recruit soluble α-Syn monomers to aggregate and propagate the structural information to the α-Syn monomers.

To explore whether the PS2 fiber exhibits detectable higher cytotoxicity than the WT2 fiber, the fibers (1 μM based on the monomer) were added extracellularly to human neuroblastoma SH-SY5Y cells, and the cell viability was measured with the MTT assay. The PS2 fiber showed higher cytotoxicity compared to the WT2 fiber, as the PS fiber did (Fig. 4e). We used mouse N2a cells to perform the same analysis, with the same results (Supplementary Fig. S3B).

The WT and PS fibers have different structural characteristics. They can recruit α-Syn monomers to form a WT2 fiber and a PS2 fiber, respectively, which share the same structural features as their respective preformed fibers. Moreover, the PS2 fiber induces higher cytotoxicity than the WT2 fiber, just as the PS fiber does relative to the WT fiber. All of these observations strongly suggest that the two preformed fibers that we have generated are different α-Syn strains and that phosphorylation induced distinct α-Syn strain formation: the WT strain and the PS strain.

### Propagation of WT and PS strains in mammalian cells

**Figure 5:**
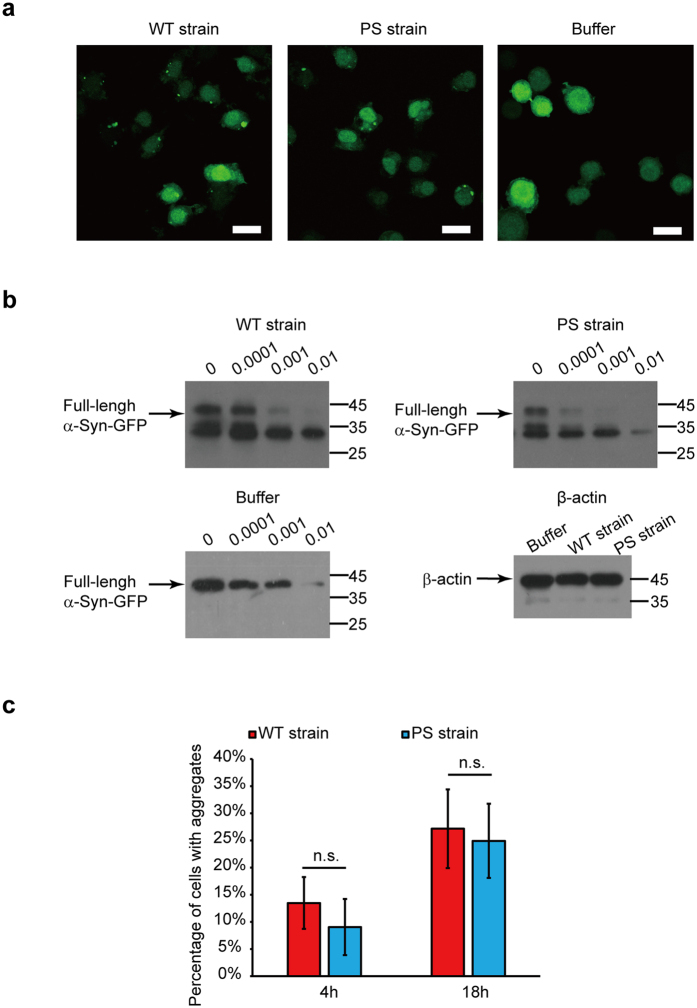
Propagation capacities of WT strain and PS strain in mammalian cells. (**a**) Confocal images of N2a cells stably expressing GFP-tagged α-Syn treated with 1 μM WT and PS strains, respectively, for 18 h. Both strains induced intracellular foci formation. The confocal image of cells untreated with α-Syn strains was used as control; the intracellular α-Syn-GFP remained diffuse. Scale bar, 20 μm. (**b**) Western blot analysis of the proteolytic digestion patterns of α-Syn-GFP in N2a cells treated with 1 μM WT or PS strains for 24 h. The control cells were treated with the same volume of PBS. The cell lysate was harvested via sonication for 12 s on ice with a sonifier. Aliquots of cell lysate were exposed to increasing concentrations of PK (0.0001−0.01 μg/mL) for 10 min at 25 °C, and the degradation pattern was analyzed by western blot. The digestion patterns of α-Syn-GFP aggregates seeded by the WT strain were significantly different from those seeded by the PS strain. Both degradation patterns of α-Syn-GFP were significantly different from that of the control cells. Βeta-actin was used as a loading control. The molecular mass marker is shown on the right of each blot. (**c**) The percentage of N2a cells stably expressing GFP-tagged α-Syn with aggregates after treatment with 1 μM WT and PS strains, respectively, for 4 h or 18 h. The number of total cells and cells with aggregates were counted in a blind manner by two scientists. Values for all strains are mean ± SD (n = 3 independent measurements). Statistical significance was determined by one-way ANOVA. There is no significant difference between the two strains.

The exogenous α-Syn fiber can be transported to cultured cells via endocytosis, inducing inclusion formation by recruiting endogenous α-Syn^6^,^7^. To evaluate whether the WT and PS strains can propagate the endogenous α-Syn to assemble into aggregates, N2a cells stably expressing GFP-tagged α-Syn were exposed to the two strains. The recruitment of soluble and diffuse α-Syn-GFP to cluster together by the two strains was observed with confocal fluorescence microscopy after fixing the cells for the indicated time. Upon addition of each of the two strains for 18 h, the redistribution of α-Syn-GFP and the presence of intracellular foci was observed (Fig. 5a). In the absence of the exogenous strain, the GFP fluorescence was diffuse in the cytoplasm and nucleus (Fig. 5a). It was clear both strains induced intracellular foci formation.

To explore whether the two strains could propagate their structural information to the intracellular α-Syn, the N2a cells stably expressing GFP-tagged α-Syn were treated with the two strains (1 μM based on the monomer), respectively, for 24 h. The cells were then collected and lysed by ultrasonication and the cell lysate harvested by centrifugation and subjected to increasing PK concentrations. The western blot of α-Syn showed that the limited proteolysis of the intracellular α-Syn-GFP aggregates induced by the PS strain was less resistant to PK than that induced by the WT strain, just as the strains themselves (Fig. 5b). The observation that the PK proteolytic pattern is different between the conditions after seeding *in vitro* and *in cellulo*, might be explained by the fact that amplification was performed with a complex cellular system. Also, the difference might be partially attributed to epitope recognition of the antibody used in the western blot analysis. The degradation pattern of α-Syn-GFP was significantly different from that of cells in the absence of an exogenous strain (Fig. 5b). The results suggested that the exogenous strain imprinted its structural characteristics on the endogenous α-Syn.

To access whether one of the strains induce more aggregates than the other strain, we counted the percentage of cells exhibiting aggregates after the treatment with two strains for 18 h. The observation was that about 30% of the cells exhibit aggregates for both strains and no detectable significance between the two strains (Fig. 5c). After treatment with two strains for only 4 h, 10% of the cells with aggregates were observed (Fig. 5c). Together with all these findings, it demonstrates that WT and PS strains can propagate the endogenous α-Syn to assemble into aggregates and imprint its structural features on the endogenous α-Syn.

## Discussion

Expanding evidence supports that synucleinopathies share features similar to prion disease, especially the existence of α-Syn strains. Our findings presented here suggest that phosphorylation at Ser129 induces distinct α-Syn strain formation, providing the first evidence that the PTMs of α-Syn can induce different strain formation and increase the variety of α-Syn strains. By employing expressional chemical ligation, we synthesized homogeneous α-Syn phosphorylated at Ser129 specifically. The subsequent results demonstrated that WT and PS fibers have different structures and propagate their respective intrinsic structure faithfully through recruiting soluble α-Syn *in vitro* and intracellularly. The implication of these findings is that they are two distinct α-Syn strains, namely WT and PS. In addition, the PS strain induced much higher cytotoxicity, suggesting that the PTM status of α-Syn in the pathogenetic process may affect strain formation.

The concept of “strains” originated with prion diseases. In prion diseases, the conformational differences in infectious prion protein aggregates are associated with the diverse clinical symptoms among individuals^26^. The infectious prion particles with a specific conformation are believed to propagate throughout the brain by imprinting their intrinsic misfolded structure on the normal prion molecule, with each different structure considered to be a “strain.” Recently, increasing evidence suggests that the different synthetic α-Syn conformers behave in a fashion similar to that of prion strains. Two distinct strains of α-Syn were prepared via repetitive seeded fibrillization *in vitro* and showed different abilities to cross-seed tau aggregation in cultured neurons and *in vivo*^9^. Two different α-Syn strains, named fibers and ribbons, were generated through the regulation of solution conditions, and these α-Syn strains with different structural characteristics and seeding propensities were associated with distinct histopathological and behavioral phenotypes^10^,^11^. This pattern implies that distinct α-Syn strains form in specific cellular environments and that α-Syn fibril polymorphism may be related to the pathological process. A mutation of α-Syn, A30P, also induces distinct strain formation compared with WT α-Syn^27^. Furthermore, C-terminally truncated α-Syn can accelerate fibrillization of α-Syn^25^ and promote various aggregated strain formation^9^.

The main component of the pathological lesions is extensively phosphorylated α-Syn at Ser129 (pS129 α-Syn)^14^. To investigate whether phosphorylation promotes distinct and more toxic α-Syn strain formation, we semisynthesized site-specific homogenous pS129 α-Syn without any mutation. We prepared WT and PS fibers with WT and pS129 α-Syn monomers, respectively. The two fibers described here exhibited structural variations. The conformation of soluble monomeric α-Syn has been proposed to be maintained by intramolecular long-range N/C-terminal tertiary interactions^24^. These contacts stabilize α-Syn in an autoinhibitory conformation. Based on the antiparallel five β-strand fibrillary α-Syn model^28^, Salvatella *et al.* proposed that the transient interactions of hydrophobic regions (β4–β5 strands etc.) lead to early aggregate formation^29^. The C-terminal region (residues 104–130) contacts residues comprising β4 and β5 in monomeric α-Syn^29^, which is important in modulating the early aggregation process. The implication is that phosphorylation at Ser129 may interfere with the transient interactions, increase conformational flexibility^30^, and break the autoinhibitory conformation, which are compatible with the influence in fibrillization exerted by phosphorylation of Ser129 and could characterize structural difference between α-Syn WT and PS fibers.

Fibril structure is highly associated with cellular dysfunction^10^,^11^,^31^,^32^,^33^,^34^. The fibril structure was observed to be modulated by buffer components, pH, and temperature *et al.*^10^,^32^,^33^, which was utilized to prepare fibers with different structures and investigate structure-activity relationships. The structural flexibility and hydrophobic exposure are suggested to be the dominant determinants of aggregates causing cytotoxicity^31^,^32^, which is consistent with our observation that PS fibers showed less stability and higher cytotoxicity than WT fibers. While Melki *et al.* demonstrated that the more PK-resistant α-Syn strain is more toxic, which is in contrast to our observation^10^,^11^. The inconsistency may result from the various pathogenic mechanism of α-Syn strains.

The phosphorylation at Ser129 is demonstrated to modulate the fibril structure, propagation properties and cytotoxicity, indicating a different strain formation. The phosphorylation status of α-Syn increases the complexity and heterogeneity of the strains. Not all of the phosphorylation sites affect the fibril structure of α-Syn, however. Phosphorylation at Tyr125, for example, does not alter the morphology and structure of the α-Syn fiber^35^. The high diversity of the phosphorylation status for individuals in the pathogenetic process, which may lead to the distinct strains described here.

The higher cytotoxicity that the PS strain induced requires attention. Previous work has demonstrated that pS129 α-Syn levels increase in the midbrain of aged monkeys, related to the increased expression of Polo-like kinase 2 within the neurons^36^. Intracellular pS129 α-Syn has been reported to be predominantly deposited as insoluble aggregates in LBs in the brains of PD patients^14^,^37^. The abundance and accumulation of pS129 α-Syn in LBs compared with that in the cytosol is consistent with a hypothesis in which cytosolic pS129 α-Syn has a higher tendency to deposit in LBs. The pS129 α-Syn promotes aggregation *in vitro*^14^,^17^ and enhances inclusion formation, which additionally supports this hypothesis. How strain formation arises *in vivo* remains a mystery. We speculate that the phosphorylation at Ser129 may be sharply increased by phosphokinase upregulation and promote the more toxic PS strain formation.

Our findings strongly suggest that pS129 α-Syn induces a structurally distinct and functionally more toxic strain. The implication is that downregulation of pS129 α-Syn likely reduces the possibility of toxic strain formation. To this end, phosphorylation at Ser129 can be a therapeutic target, and regulation of pS129 α-Syn levels may represent a novel strategy for treating synucleinopathies.

## Methods

### Preparation of α-Syn phosphorylated at Ser129

The generation of α-Syn phosphorylated at Ser129 involved a NCL reaction between α-Syn(1–106)SR and synthetic peptide α-Syn (A107C–140) pS129. NCL reactions were carried out via mixing 0.8 mM of α-Syn(1–106)SR and 4.0 mM α-Syn (A107C–140) pS129 peptide in 1 mL 6 M GuHCl, 200 mM sodium phosphate buffer at pH 7.0 supplemented with 30 equiv of TCEP and MPAA for 4 h at 37 °C. A 15% SDS-PAGE was used to monitor the reaction; after 4 h, all of the α-Syn(1–106)SR transformed to the ligation product. The reaction mixture was desalted through a 5 mL HiTrap desalting column with 6.0 M GuHCl, 200 mM sodium phosphate buffer, pH 7.0, as the mobile phase to remove the MPAA. Then free-radical-based desulfurization of the pS129 α-Syn (A107C) was carried out as previously described. Briefly, 1.2 mL of the desalted fraction was mixed with a 400 μL sample of a solution of 1 M of TCEP (in 6 M GuHCl, 200 mM sodium phosphate, pH 7.0). Then a 200 μL sample of 2-methyl-2-propanethiol and 200 μL of a 0.1 M solution of 2-2’-azobis [2-(2-imidazolin-2-yl)propane] dihydrochloride (VA-044) (in 6 M GuHCl, 200 mM sodium phosphate, pH 7.0) were added under argon. The reaction was incubated at 37 °C overnight.

The semisynthetic pS129 α-Syn was finally purified using preparative HPLC (Shimadzu, LC-6AD) with a Proteonavi column (Shiseido, 5 μm, 10 × 250 mm) with a linear gradient of 20−70% B for 30 min at a flow rate of 10 mL/min (solvent A was water/0.06% TFA and solvent B was 80% acetonitrile/20% water/0.06% TFA). The purified pS129 α-Syn was lyophilized and stored at −80 °C until use. The final yield of pure lyophilized protein was around 26% (3 mg). The purity of the pS129 α-Syn preparation (>95%) was assessed by 15% SDS-PAGE, analytical RP-HPLC, and mass spectrometry analysis.

### Assembly of α-Syn into fibers

α-Syn fibers were prepared by incubating purified WT α-Syn and pS129 α-Syn (1 mg/mL in PBS) at 37 °C in the presence of a glass bead with constant agitation for 1 week. The Eppendorf Thermomixer was set at 600 rpm.

### Aggregation kinetics of α-Syn *in vitro*

The kinetics of aggregation of α-Syn was measured in low binding, clear-bottomed 96-well plates (Corning, cat. no. 3603) with plate reader (Bioteck, Synergy 4 Plate Reader) in bottom reading mode. 100 μL PBS containing 10 μM Thioflavine T and 40 μM α-Syn was incubated in the plates with constant shaking. Each reading cycle was followed by 10 min of shaking. The measurement of the propagation activities of α-Syn fibers *in vitro* was performed with 35 μM α-Syn in the absence or presence of 3.5 μM preformed α-Syn fibers incubated in the plates under constant shaking condition. To identify the dominance of the two fibers, the measurement of the kinetics of aggregation of WT monomeric α-Syn in the presence of fibers was performed under quiescent condition. For the co-incubation with both fibers, WT and PS fibers (3.5 μM for each fiber) were added to 35 μM α-Syn.

### Transmission electron microscopy

α-Syn fibers (7 μM) were incubated on the copper grids for 90 s. The grids were negatively stained with 2% sodium phosphotungstic acid for 60 s after the sample adsorption. The grids were then air-dried and the samples were examined using a Hitachi-7650B electron microscope at 80 kV.

### ThT fluorescence

The ThT fluorescence (excitation, 440 nm; emission, 480 nm) was measured in 96-well plates (Corning, cat. no. 3915) with a plate reader (Bioteck, Synergy 4 Plate Reader). The final concentration of ThT and α-Syn fibers was 10 μM and 3.5 μM, respectively.

### X-ray diffraction

The fibers were washed with water by repeated centrifugation. The droplets of fiber suspension were aligned between the ends of two glass capillaries and dried. The X-ray diffraction experiments were performed at room temperature on a Rigaku MicroMax007HF generator. Data were collected with a sample-detector distance of 60 mm and with 60 s exposure, and diffraction was observed at 2.56 Å resolution. Data were processed with HKL-3000.

### Proteolytic digestions

The aliquots of α-Syn fibers (30 μM) in PBS were treated at 25 °C by PK (0.1 μg/mL, Aladdin). The reactions were stopped at different time intervals by addition of sample buffer (50 mM Tris-HCl, pH 6.8, 10% glycerol, 2% SDS, 1% β-mercaptoethanol and 0.1% bromophenol blue) and keeping the Eppendorf tube in boiled water immediately. After treatment of each tube for 10 min, the samples were separated on 15% SDS-PAGE to monitor the cleavage pattern of α-Syn.

### Cytotoxicity of *in vitro* α-Syn fibers

N2a cells were cultured in Dulbecco’s modified Eagle’s medium/minimal Eagle’s medium 1:1 supplemented with 10% fetal bovine serum and were plated at a density of 6,000 cells per well in 150 μL medium. SH-SY5Y cells were cultured in Roswell Park Memorial Institute (RPMI) 1640 supplemented with 10% fetal bovine serum and were plated at a density of 9,000 cells each well in 150 μL medium. Cells were cultured in 96-well plates (Corning, cat. no. 3695) for 18 h in cell incubator at 37 °C in 5% CO2 before addition of fibers. 140 μL of fresh medium containing indicated concentration of α-Syn fibers was added to each well and incubated for 24 h in cell incubator. Control samples were prepared with the addition of identical volumes of buffer. Then, 15 μL MTT (5 mg/mL) was added into each well, followed by incubation for another 4 h in cell incubator. The medium was drained away with syringe and the formazan was dissolved with 150 μL DMSO followed by incubation for 10 min at 37 °C. The absorbance was measured at 570 nm.

The measurement of caspase-3 activity was performed with the Caspase 3 Activity Assay Kit (Beyotime, C1115) according to the manufacturer’s instructions. SH-SY5Y cells were treated with α-Syn fibers for 24 h and cell lysis buffer (Beyotime, C1115-1) was added to the cells and the extracts were incubated with the substrate of caspase 3(acetyl-Asp-Glu-Val-Asp p-nitroanilide) for 2 h at 37 °C. The absorbance at 405 nm of the p-nitroaniline formed upon cleavage of caspase-3 was measured with a plate reader (Bioteck, Synergy 4 Plate Reader).

For the intracellular ROS levels measurement, the fluorescent probe 2′,7′-Dichlorofluorescin diacetate (DCFH-DA) was used. The SH-SY5Y cells were plated on the 96-well plates (Corning, cat. no. 3603) and cultured for 24 h. After treatment with 1 μM α-Syn fibers for 1 h, the fresh medium containing 2.5 μM DCFH-DA was added to the cells and incubated for 30 min. Followed by three times of washes with PBS, the fluorescence intensity was measured with the excitation and emission wavelengths set at 488 and 525 nm respectively.

For calcein release, the lipid films were resuspended in PBS containing 70 mM calcein and treated as described above. After extrusion, the vesicle loaded with calcein was purified with PD-10 desalting columns (GE Healthcare). Calcein-capculed lipid vesicles were incubated with 10 μM a-syn fibers in the 96-well plates (Corning, cat. no. 3915) and the calcein release were monitored for 1 h with the fluorescence increase (excitation, 490 nm; emission, 520 nm). The 100 percent of vesicle disruption was performed upon addition of 0.1% Triton X-100 at the end of each experiment.

### Intracellular propagation activities of α-Syn fibers

To construct the stable cell lines overexpressing α-Syn-GFP, a plasmid was made by PCR amplification of WT α-Syn cDNA and inserting into pEGFP-N3 vector between the XhoI and SmaI sites followed by EGFP sequence. The primers sequence were as follows: forward 5′-CCG CTC GAG ATG GAT GTA TTC ATG AAA GGA CT-3′, reverse 5′-TCC CCC GGG ATG GCT TCA GGT TCG TAG TCT TGA-3′. Transfection was performed with Lipofectamin2000 following the manufacturer instruction. Stable cell lines expressing α-Syn-GFP were selected with 800 μg/mL neomycin (G418) for 3 weeks, and sorted on the basis of their fluorescent intensity of GFP by FACS (BD Biosciences, FACSAria III). N2a cells stably expressing α-Syn-GFP were plated on the poly-L-lysine-coated coverslips and grown for 18 h before the addition of α-Syn fibers. The cells were exposed to 1 μM α-Syn fibers for 18 h. Then the cells were washed with PBS for 3 times and fixed with 4% paraformaldehyde. Cells with intracellular foci were observed in randomly selected fields by laser scanning confocal microscope (Zeiss, LCM780).

Proteolytic digestions of cell extracts. N2a cells stably expressing α-Syn-GFP were plated on 21 cm^2^ dishes followed by treating with 1 μM α-Syn fibrils for 24 h. The cells were lifted with trypsin and washed with PBS. The final density of the cells was 5 × 10^6^ cell/mL. The cells were sonicated for 12 s on ice with sonifier. Aliquots of cell lysate were exposed to increasing concentrations of PK (0.0001−0.01 μg/mL) for 10 min at 25 °C. The samples were then treated as demonstrated above and analyzed on 15% SDS-PAGE followed by western-blot using mouse anti-α-Syn ab27766 (1:1000 dilution, Abcam) to detect remaining WT α-Syn.

## Additional Information

**How to cite this article**: Ma, M.-R. *et al.* Phosphorylation induces distinct alpha-synuclein strain formation. *Sci. Rep.* **6**, 37130; doi: 10.1038/srep37130 (2016).

**Publisher’s note:** Springer Nature remains neutral with regard to jurisdictional claims in published maps and institutional affiliations.

## Supplementary Material

Supplementary Information
