# Supplementary material for: Phosphorylation induces distinct alpha-synuclein strain formation
Source: Sci Rep. 2016 Nov 17;6:37130. doi: 10.1038/srep37130 (PMC5112567; doi:10.1038/srep37130)
Supplement: Supplementary Information [file srep37130-s1.doc]

**Supplementary Information for**

**Phosphorylation induces distinct alpha-synuclein strain formation**

**Meng-Rong Ma^1^, Zhi-Wen Hu^1^, Yu-Fen Zhao^1^, Yong-Xiang Chen^1^, Yan-Mei Li^1, 2,*^**

**1- Department of Chemistry, Key Laboratory of Bioorganic Phosphorus Chemistry & Chemical Biology (Ministry of Education),Tsinghua University, 100084 Beijing, P. R. China**

**2- Beijing Institute for Brain Disorders, 100069 Beijing, P. R. China**

**Supplementary Methods**

**Synthesis of α-Syn(A107C−140) pS129 peptide.** The peptide fragment α-Syn(A107C−140) phosphorylated at Ser129 was synthesized through ge-neral Fmoc solid-phase synthesis strategy. Generally, the synthesis of the peptide was carried out on preloaded Fmoc-Ala-Wang Resin (0.18 mmol/g, 0.3 mmol scale). The peptide coupling reaction was performed in the presence of 4 equiv of Fmoc-amino acids, 3.6 equiv of 1-[Bis(dimethylamino)methylene]-1H-1,2,3-triazolo[4,5-b]pyridinium 3-oxid hexafluorophosphate (HATU), 4 equiv of 1-Hydroxy-7-azabenzotriazole (HOAt) and 8 equiv of N,N-diisopropylethylamine (DIEA) with the resin for 1 h at room temperature. Fmoc-Ser[PO(OBzl)]-OH building block was used to bring in the phosphorylated serine at 129. The Fmoc-group was deprotected by treating the resin with 20 % piperidine in N,N-dimethylformamide (DMF). The final deprotections of peptide side chain and cleavage of peptide from the resin were performed through addition of the mixture of TFA/water/phenol/thioanisole/1,2-ethanedithiol(EDT)/Me2S/NH4I (81/5/3/5/2.5/2/1.5) for 3.5 h. After the filtration and removal of TFA, the peptide was precipitated in cold ether drop-wisely. The preparative HPLC (Shimadzu, LC-6AD) was used to purify the crude peptide using reversed-phase C18 column (YMC, 5 μm, 20×250 mm) with a linear gradient of 20−70% B for 30 min at a flow rate of 10 mL/min (solvent A was water/0.06% TFA and solvent B was 80% acetonitrile/20% water/0.06% TFA). The peptide was identified with ESI-MS analysis and the peptide purity was confirmed with analytical RP-HPLC (Shimadzu, LC-2010A HT) using C18 column (YMC, 5 μm, 4.6×150 mm) with the same gradient of preparative HPLC at a flow rate of 0.8 mL/min.

**Expression and purification of recombinant α-Syn(1−106)SR.** The human α-Syn(1−106) fragment was inserted into the pTWIN vector containing intein and a CBD using the following primers: forward 5’-GGA ATT CCA TAT GGA TGT ATT CAT GAA AGG ACT TT-3’ and reverse 5’-GGA CTA GTG CAT CTC CCG TGA TGC ATC CTT CTT CAT TCT TGC CCA-3’. The α-Syn(1−106) fragment fused with intein and CBD was expressed in *E. coli* BL21(DE3). Protein expression was induced by addition of 1 mM isopropyl-β-D-1-thiogalactopyranoside (IPTG) and incubation of cells for 18 h at 16 °C. The cells were harvested by centrifugation and resuspended in lysis buffer (20 mM HEPES, 500 mM NaCl, pH 8.0, supplemented with 100 μg/mL phenylmethanesulfonyl fluoride (PMSF) and 0.1% Triton). The cells were lysed by sonication, and the supernatant was separated from the cell debris by centrifugation (25,000 ×g, 60 min, 4 °C). The supernatant was loaded on the chitin beads pre-equilibrated with 20 mM HEPES and 500 mM NaCl at pH 8.0. A wash step was performed with lysis buffer for four column volumes and lysis buffer without Trion for another eight column volumes to remove the nonspecific binding. The splicing reaction was carried out by the addition of 0.25 M MESNa in the eluate and incubation of the column for 12 h at 4 °C. The elution fraction, α-Syn(1−106)SR, was analyzed by SDS-PAGE and concentrated by ultrafiltration. The α-Syn(1−106)SR was stored in 20 mM HEPES, 150 mM NaCl, 50 mM MESNA, pH 8.0, at −80 °C until use. The purity of α-Syn(1−106)SR was around 90%. The final yield of α-Syn(1−106)SR was 8 mg/L of BL21(DE3) expression medium.

**Expression and purification of recombinant WT α-Syn.** The plasmid pET22b encoding WT α-Syn was constructed using the following primers: forward 5’-GGA ATT CCA TAT GGA TGT ATT CAT GAA AGG ACT-3’, reverse 5’-CCG CTC GAG CTA TTA GGC TTC AGG TTC GTA GTC-3’. The *E. coli* BL21(DE3) was transformed with the plasmid. The expression and extraction of recombinant WT α-Syn was done as described previously by Wang et al.[^1^](#_ENREF_1). The supernatant from the osmotic shock treatment containing α-Syn was further purified with preparative HPLC (Shimadzu, LC-6AD) using Proteonavi column (Shiseido, 5 μm, 10×250 mm) with a linear gradient of 20−70% B for 30 min at a flow rate of 10 mL/min (solvent A was water/0.06% TFA and solvent B was 80% acetonitrile/20% water/0.06% TFA). The purified WT α-Syn was lyophilized and stored at −80 °C until use. The purity of the pS129 α-Syn preparation (>95%) was assessed by 15% SDS-PAGE, analytical RP-HPLC and mass spectrometry analysis. The final yield of recombinant WT α-Syn was 10 mg/L of BL21(DE3) expression medium.

**Western blot analysis.** 1 μg WT α-Syn and pS129 α-Syn were separated on 15% SDS-PAGE and transferred onto a piece of polyvinylidene fluoride (PVDF) membrane (Millipore) using a semidry transfer system (Bio-Rad) under constant voltage (30V) for 40 min. After blocking with 5% BSA (diluted with 50 mM Tris, 150 mM NaCl, 0.05% Tween 20, pH7.4; TBST) at room temperature for 1 h, the membranes were incubated with the respective primary antibodies overnight at 4 °C: mouse anti-α-Syn ab27766 (1:1000 dilution, Abcam), rabbit anti-α-Syn ab51252 (1:1000 dilution, Abcam). After three washes with TBST, the membrane was incubated with HRP-conjugated secondary rabbit anti-mouse or goat anti-rabbit antibodies (1:2000 dilution, Invitrogen). Followed by three washes with TBST, the bands were visualized with the Chemiluminescent Substrate (Thermo), and the images were captured with Medical X-ray Processor (Kodak).

**Far-UV CD spectra.** The spectra of α-Syn were measured in the absence and in the presence of lipid vesicles using the Chirascan plus CD spectrophotometer (Applied Photophysics). The final concentration of protein was 20 μM (diluted in PBS) and the lipid-protein mole ratio of 10:1. The spectra were recorded in 1 mm path length cuvette with a scan range of 195–260 nm. The final spectra were averaged over 3 scans and were buffer subtracted and smoothened using Pro-Data software provided with the instrument. The lipid vesicles preparation was carried out as described previously. Briefly, the POPG was dried by a stream of nitrogen and desiccated by vacuum to remove the organic solvent. Then PBS was added to hydrate the lipid film. After five times of freeze-thaw cycles, the solution was extruded through a 100 nm polycarbonate membrane.

**Reference**

1 Huang, C., Ren, G., Zhou, H. & Wang, C. C. A new method for purification of recombinant human alpha-synuclein in Escherichia coli. *Protein Expr. Purif.* **42**, 173-177 (2005).


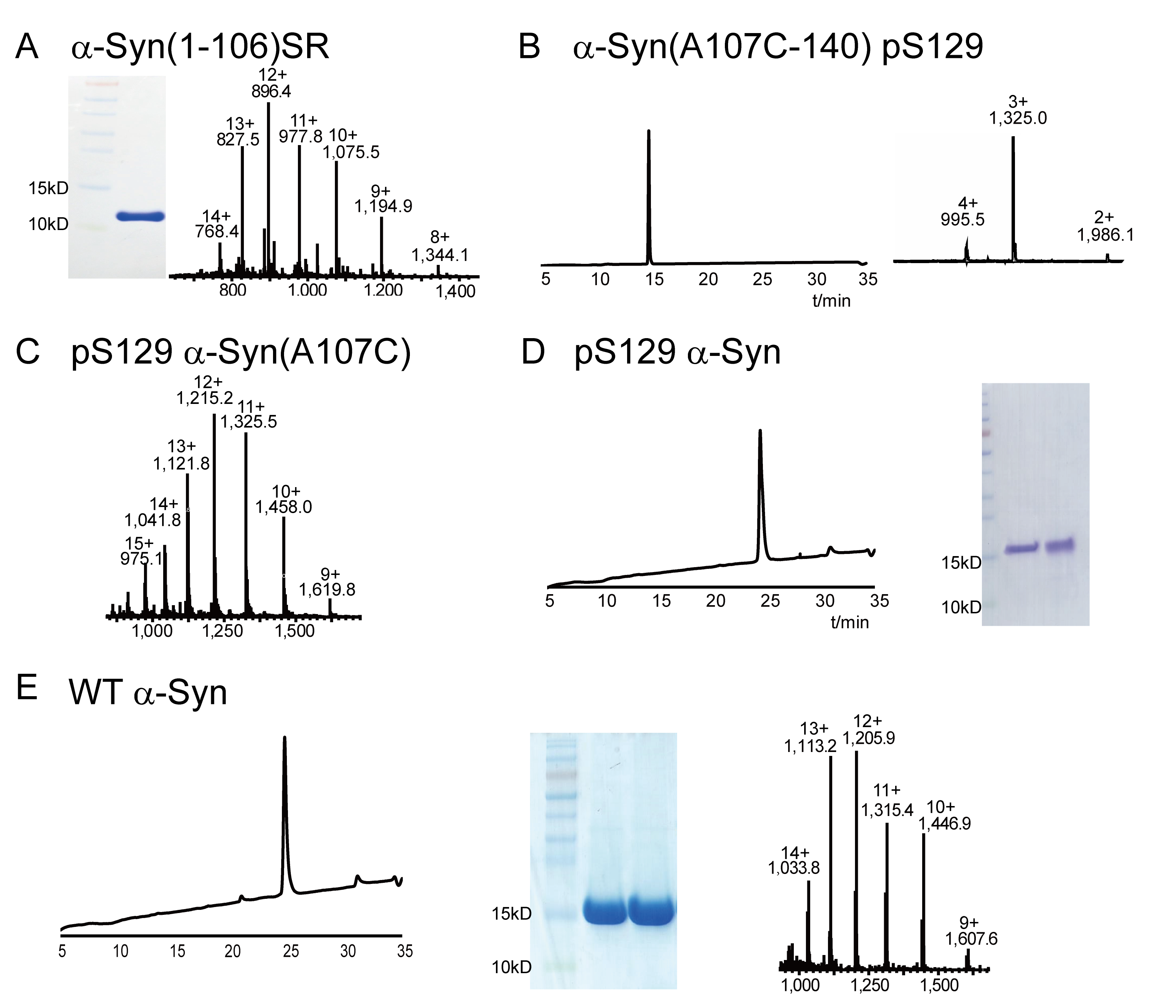


**Supplementary Figure S1: Characterization of α-Syn(1−106)SR, α-Syn(A107C−140) pS129, pS129 α-Syn(A107C), pS129 α-Syn and WT α-Syn.**

**A,** SDS-PAGE and ESI-MS analysis of α-Syn(1−106)SR. The observed mass of 10,744.8 Da is consistent with the calculated mass of 10,743.3 Da.

**B,** RP-HPLC and ESI-MS analysis of α-Syn(A107C−140) pS129. The observed mass of 3,970.2 Da is consistent with the calculated mass of 3,969.0 Da.

**C,** ESI-MS analysis of pS129 α-Syn(A107C) before desulfurization. The observed mass of 14,570.4 Da is consistent with the calculated mass of 14,570.2 Da.

**D,** RP-HPLC and SDS-PAGE analysis of pS129 α-Syn.

**E,** RP-HPLC, SDS-PAGE and ESI-MS analysis of WT α-Syn. The observed mass of 14,458.8 Da is consistent with the calculated mass of 14,460.1 Da.


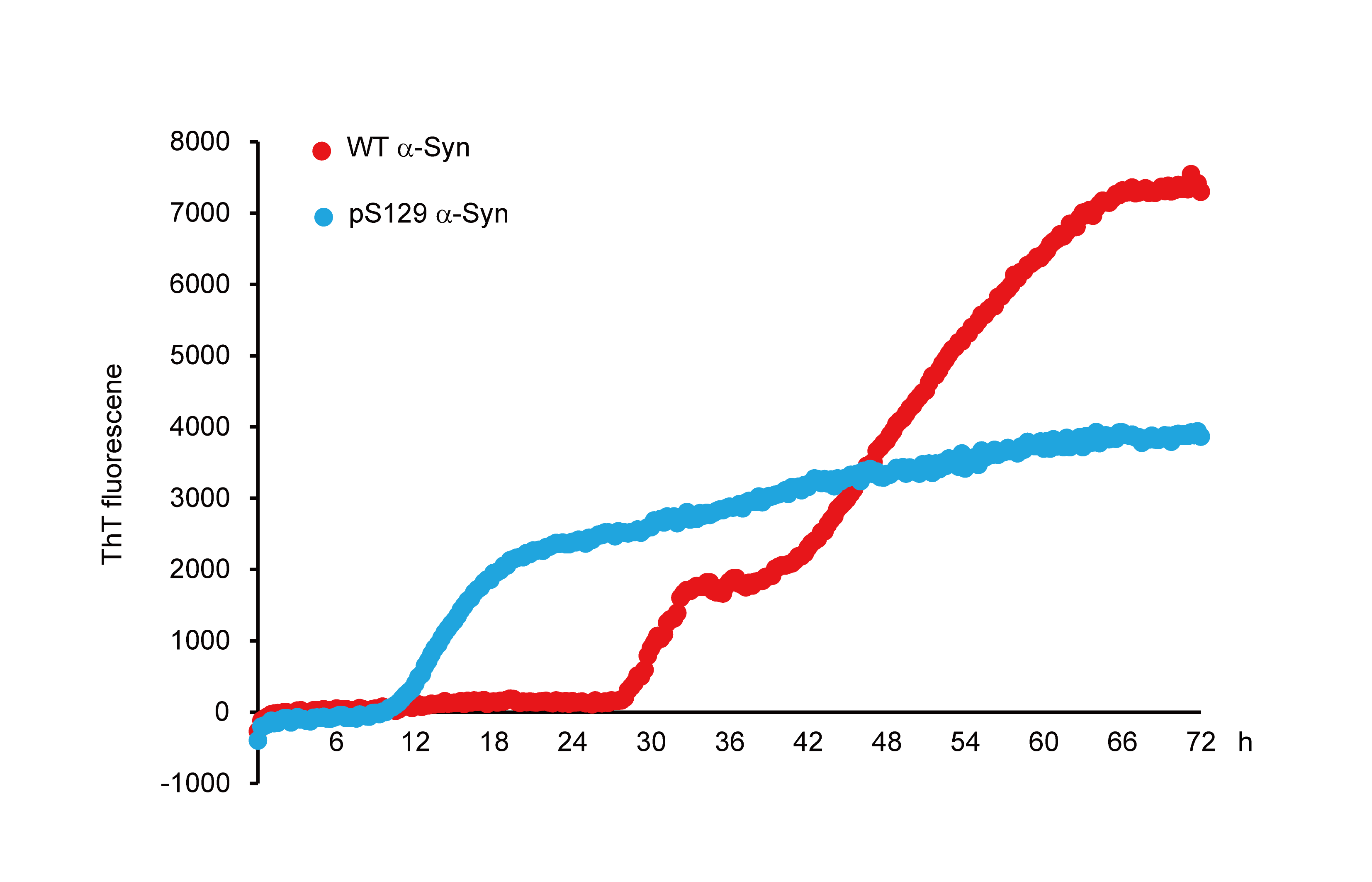


**Supplementary Figure S2: The kinetics of aggregation of WT and pS129 α-Syn.**

Fibrillization kinetics of 40 μM WT (red dot) and pS129 (blue dot) α-Syn at 37°C was monitored by ThT fluorescence. Data were obtained by subtracting the identical concentration of ThT solution.


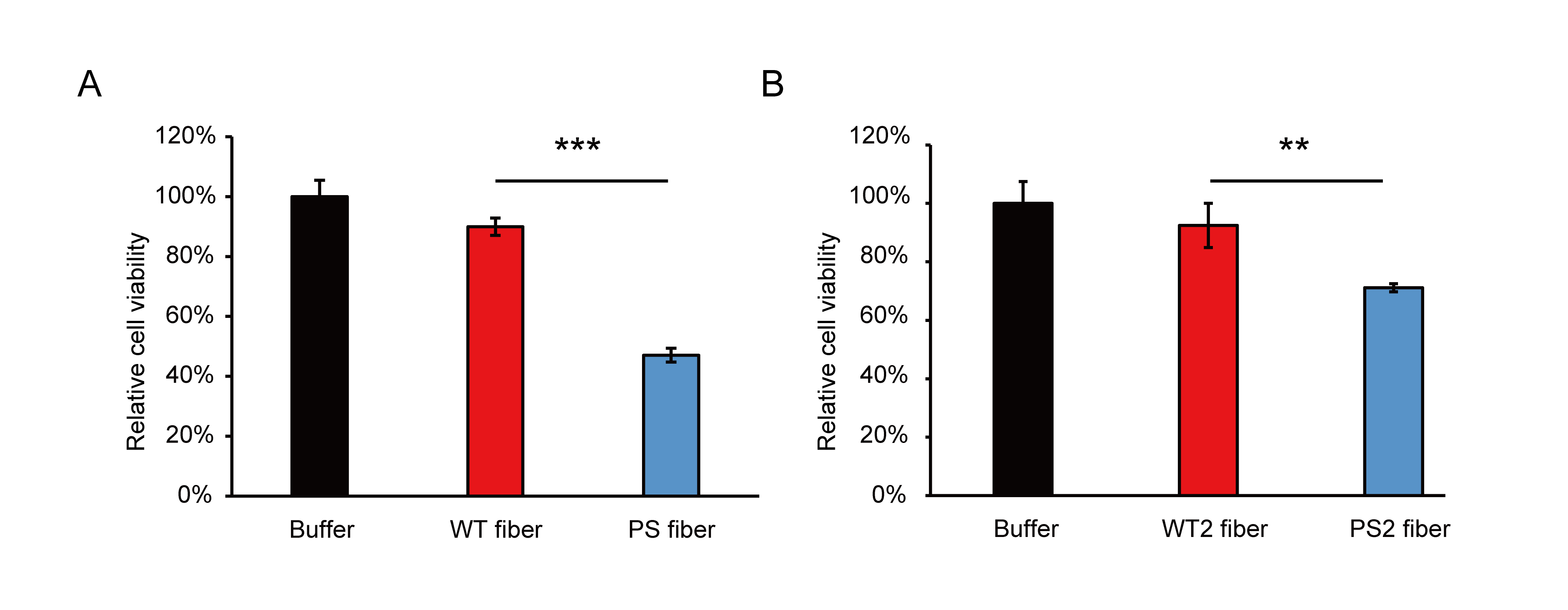


**Supplementary Figure S3: Toxicity of WT, PS, WT2, and PS2 fibers.**

**A,** Cell viability of N2a cells measured by MTT assay. N2a cells were treated with buffer (Buffer, black bar), 0.1 μM WT fiber (red bar), or 0.1 μM PS fiber (blue bar) for 24 h.

**B,** Cell viability of N2a cells measured by MTT assay. N2a cells were treated with buffer (Buffer, black bar), 0.1 μM WT2 fiber (red bar), or 0.1 μM PS2 fiber (blue bar) for 24 h. Values for all fibers are mean±SD (n=3 independent measurements) shown as percentages relative to buffer. Statistical significance was determined by one-way ANOVA, **P<0.01, ***P<0.001. (A) P =0.0001, α-value: 0.05; (B) P =0.0066, α-value: 0.05.

*
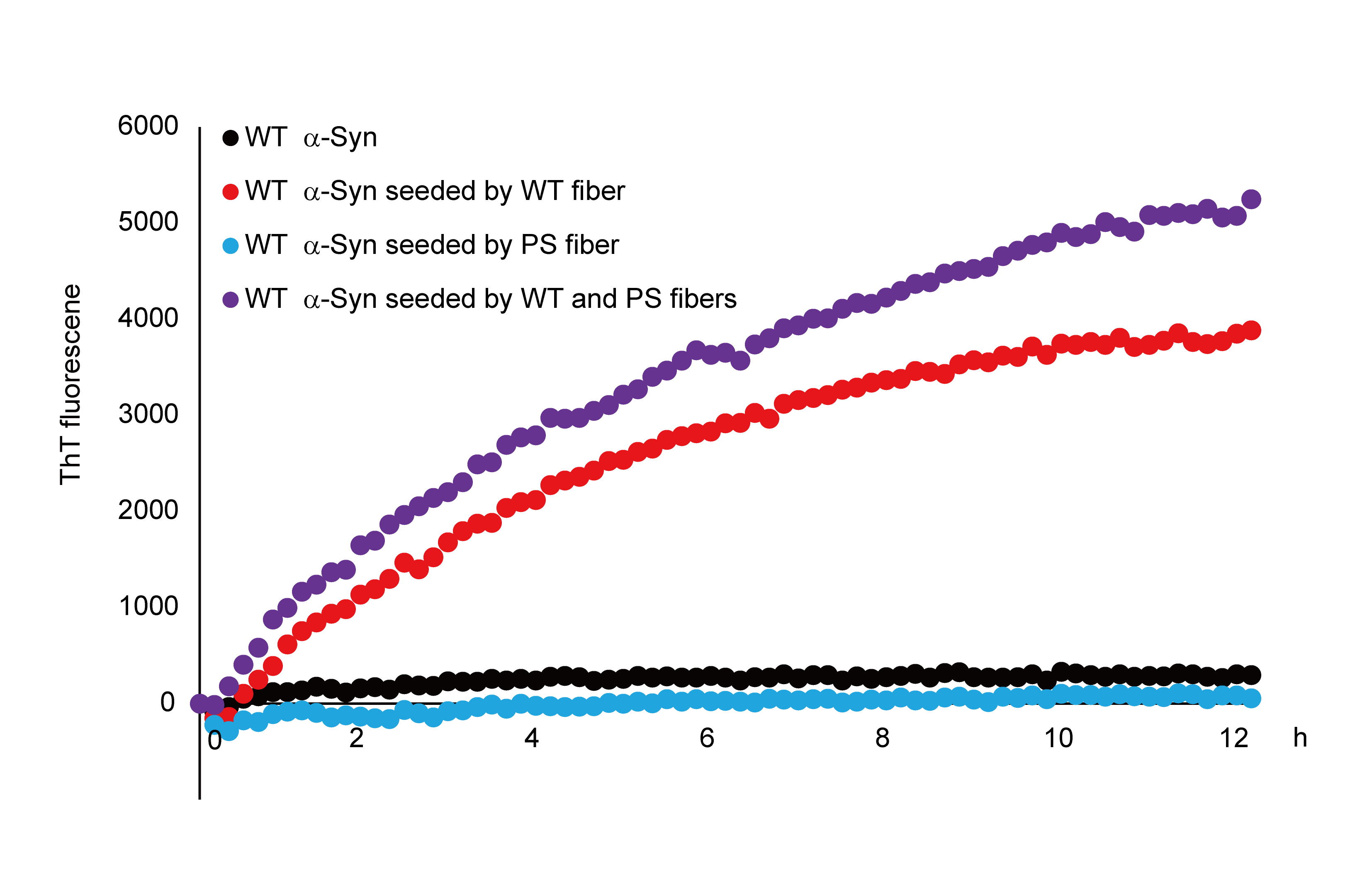
*

**Supplementary Figure S4:** The kinetics of aggregation of WT monomeric α-Syn in the presence of fibers under quiescent condition.

Fibrillization kinetics of 35 μM WT monomeric α-Syn in the absence of preformed fiber (black dot) or presence of WT fiber (3.5 μM based on monomer, red dot) or PS fiber (3.5 μM based on monomer, blue dot) or both fibers (3.5 μM WT and PS fibers respectively, purple dot) monitored by ThT fluorescence. Data were obtained by subtracting the identical conc
